# Varix of the Tongue

**Published:** 1891-06-27

**Authors:** 


### VARIX OF THE TONGUE.

A varicose condition of the veins at the root of the tongue is commonly found when sought for in a large proportion of patients who suffer from symptoms of relaxed throat. In long-standing cases a hypertrophy of the papillæ at the back of the tongue generally arises as the result of the chronic congestion. This is a condition of affairs which has been generally overlooked, although the congested fauces and pharynx have received great attention. On looking into the mouth the condition of the fauces and pharynx are most apparent, but in order to observe the condition of the veins and papillæ at the back of the tongue the laryngeal mirror must be used. The veins will then be seen very much congested and enlarged, lying between the hypertrophied adenoid tissue.

Mr. Lennox Browne was the first to direct attention to the great importance of this condition about ten years ago, and since then it has formed the subject of several contributions by specialists.

The symptoms that arise are often most distressing, and are altogether disproportionate to the apparent triviality of the cause. Mr. Lennox Browne lays stress on what he terms "faucial tenesmus," a contraction of the constrictors of the pharynx and of the faucial muscles, producing gagging whenever the parts are touched, and causing difficulty in swallowing. The patient generally suffers from darting and dragging pains in the throat and shooting up to the ears, especially on eating.

The local causes of tenesmus of the throat, writes Mr. Browne, may be arranged under three headings: (*a*) those cases in which there is really a foreign body, such as a fish-bone or plate of false teeth impacted in some part of the throat; (*b*) cases in which there is some obvious hypertrophic lesion, especially of the lymphoid tissues of the fauces or pharynx, congestion, with or without enlargement of the thryroid gland; (*c*) those cases in which there is no obvious lesions, and which are essentially neurotic.

